# The Mammoth Cave a Winter Resort for Invalids

**Published:** 1843-01

**Authors:** 


### THE MAMMOTH CAVE A WINTER RESORT FOR INVALIDS.

We understand that our enterprising friend, Dr. Croghan, continues unremitting in his efforts, to make this celebrated cavern a comfortable winter residence, for persons affected with pulmonary disease, and who are unable in autumn to migrate to the distant South. We have been told of a medical gentleman, who spent several months within it, and came out greatly relieved of a pulmonary disorder—the particular kind was not mentioned to us. We hope that other physicians, who may labor under affections of the lungs, and do not reside at inconvenient distances, will be induced to try its effects. Patients not of the profession, need not hesitate to go thither, on account of its involving a separation from their physicians, as Dr. Croghan spends most of his time there, and is well qualified to give them advice, although not now in the practice of his profession. D.

